# A Leaking-Proof Theranostic Nanoplatform for Tumor-Targeted and Dual-Modality Imaging-Guided Photodynamic Therapy

**DOI:** 10.34133/bmef.0015

**Published:** 2023-03-30

**Authors:** Duo Jin, Yang Zhu, Manman Liu, Wenxin Yu, Jiaji Yu, Xinwei Zheng, Lulu Wang, Yun Wu, Kaiju Wei, Junjie Cheng, Yangzhong Liu

**Affiliations:** ^1^ Department of Chemistry, University of Science and Technology of China, Hefei 230001, China.; ^2^ High Magnetic Field Laboratory, Chinese Academy of Sciences, Hefei 230031, China.; ^3^ Nano Science and Technology Institute, Suzhou Institute for Advanced Study, University of Science and Technology of China, Suzhou 215123, China.

## Abstract

*Objective*: A protein-based leaking-proof theranostic nanoplatform for dual-modality imaging-guided tumor photodynamic therapy (PDT) has been designed.

*Impact Statement*: A site-specific conjugation of chlorin e6 (Ce6) to ferrimagnetic ferritin (MFtn-Ce6) has been constructed to address the challenge of unexpected leakage that often occurs during small-molecule drug delivery.

*Introduction*: PDT is one of the most promising approaches for tumor treatment, while a delivery system is typically required for hydrophobic photosensitizers. However, the nonspecific distribution and leakage of photosensitizers could lead to insufficient drug accumulation in tumor sites.

*Methods*: An engineered ferritin was generated for site-specific conjugation of Ce6 to obtain a leaking-proof delivery system, and a ferrimagnetic core was biomineralized in the cavity of ferritin, resulting in a fluorescent ferrimagnetic ferritin nanoplatform (MFtn-Ce6). The distribution and tumor targeting of MFtn-Ce6 can be detected by magnetic resonance imaging (MRI) and fluorescence imaging (FLI).

*Results*: MFtn-Ce6 showed effective dual-modality MRI and FLI. A prolonged in vivo circulation and increased tumor accumulation and retention of photosensitizer was observed. The time-dependent distribution of MFtn-Ce6 can be precisely tracked in real time to find the optimal time window for PDT treatment. The colocalization of ferritin and the iron oxide core confirms the high stability of the nanoplatform in vivo. The results showed that mice treated with MFtn-Ce6 exhibited marked tumor-suppressive activity after laser irradiation.

*Conclusion*: The ferritin-based leaking-proof nanoplatform can be used for the efficient delivery of the photosensitizer to achieve an enhanced therapeutic effect. This method established a general approach for the dual-modality imaging-guided tumor delivery of PDT agents.

## Introduction

Photodynamic therapy (PDT) is a noninvasive therapeutic modality for tumor treatment, which involves the activation of photosensitizers (PSs) by laser irradiation to convert oxygen from the ground state into the cytotoxic singlet state (^1^O_2_) [1,2]. PDT has achieved great success in the clinic for solid tumor treatment, as this irradiation-based therapy can be precisely controlled in order to minimize side effects to healthy tissues [3]. However, several drawbacks, such as the low solubility of PSs and poor tumor specificity, hamper the further application of PSs [4,5]. Given these difficulties, using nanocarriers for the specific delivery of PSs with active tumor targeting could promote therapeutic effects by improving the biodistribution and bioavailability of PSs and diminishing the adverse side effects to healthy tissues [6–9].

Proteins, as a type of natural bio-macromolecules, possess inherent superiorities as delivery vectors, including excellent biocompatibility and biodegradability, which can improve the efficacy of hydrophobic drugs [10]. Among the various protein carriers, ferritin (Ftn) has gained great attention due to its hollow cage-like structure and high stability [11]. The reversible disassembly/reassembly of the cage structure of Ftn allows convenient encapsulation of therapeutic agents into the cavity of the protein [12,13]. In addition, Ftn is the major iron storage protein in mammals that can store up to 4,500 iron atoms in a nano-cluster format, which makes Ftn a suitable biomineralization template to synthesize ferrimagnetic nanoplatform [14] for diagnostic and therapeutic applications [15–17]. Notably, human Ftn possesses high affinity to transferrin receptor 1 (TfR1), a receptor overexpressed in many tumor cells. Hence, Ftn has been recently investigated for the targeted delivery of antitumor drugs [18], PSs [19], and imaging agents [20,21].

Unexpected leakage often occurs in the delivery of small molecular drugs, which could cause side effects and is problematic for further clinical development [22,23]. This is also a challenge for the Ftn-based delivery [24]. Although drug release is required for ordinary drugs, PSs can execute their function on vectors without release. Hence, PSs can be delivered through covalent conjugation to vectors. To achieve this purpose, an engineered Ftn was prepared by fusing 2 additional lysine residues at its N-terminus with a flexible (GS)_3_ linker, generating a ^KK^Ftn construct for covalent ligation of PS molecules. These 2 lysine residues are more reactive than other lysine residues due to their high accessibility on the surface of Ftn. Hence, ^KK^Ftn allows site-specific conjugation to the 2 lysine residues introduced to the protein surface. This approach minimizes the risk of disrupting the structure and function of Ftn and avoids premature release of the loaded cargoes. Notably, PSs that localize on the surface of Ftn could functionalize efficiently in killing tumor cells. As the lifetime of ^1^O_2_ in water is very short (approximately 3 μs) [25], PSs bound on the surface of carriers can be more effective than encapsulated PSs because of the advantage in the diffusion of ^1^O_2_ [26].

**Fig. 1.**
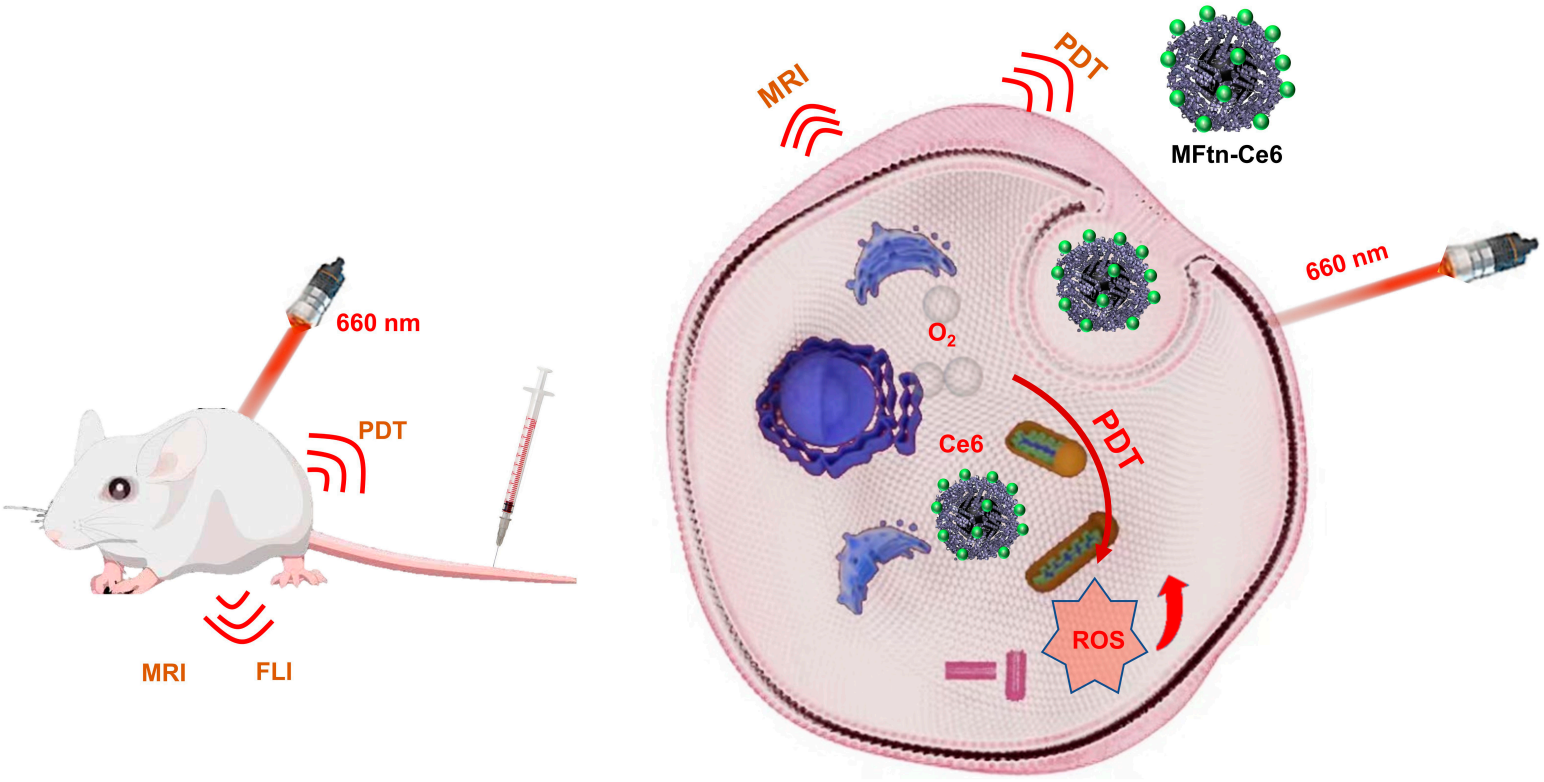
Schematic illustration of the preparation and combined therapeutic effect of MFtn-Ce6.

In order to verify the leaking-proof effectiveness of the strategy above, the iron oxide nanoparticles were encapsulated in situ in the cavity of Ftn to obtain the ferrimagnetic Ftn (MFtn), which can be easily tracked using magnetic resonance imaging (MRI). Meanwhile, chlorin e6 (Ce6), a widely used PS in PDT, has been covalently conjugated to the surface of MFtn (termed MFtn-Ce6, Fig. 1) for tumor therapy. Benefiting from the fluorescence of Ce6, the real-time biodistribution of PSs can be monitored by fluorescence imaging (FLI). This dual-modality imaging allows crosschecking of the in vivo stability and biodistribution of MFtn-Ce6, as Ce6 and iron oxide core are located in different parts of the nanoplatform. In a tumor-bearing mouse model, both the signals of FLI and MRI can be clearly observed at the tumor site even at 48 h post-injection, showing the stability and persistent tumor accumulation of this system. Guided by the dual-modality imaging results, the PDT can be applied at the proper time for efficient treatment with minimal effects on healthy tissues. In comparison to the free Ce6, MFtn-Ce6 shows more pronounced anti-tumor efficacy due to its advantages in biodistribution, circulation, and metabolism.

## Results

### Preparation and characterization of MFtn-Ce6

**Fig. 2.**
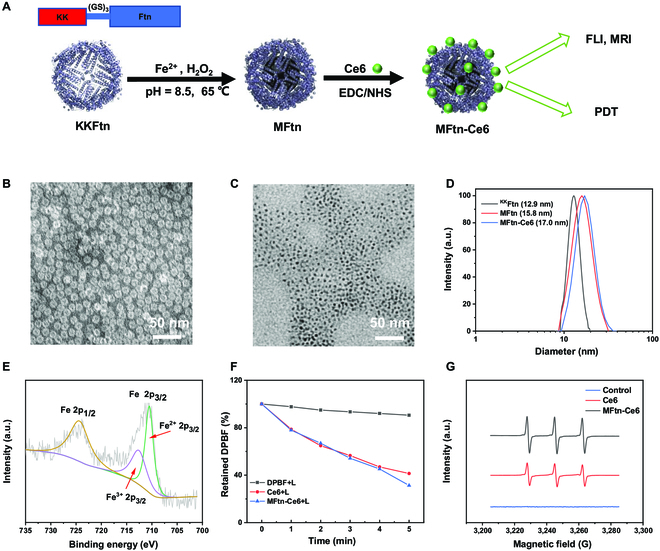
Preparation and characterization of MFtn-Ce6. (A) Schematic illustration of the procedure of MFtn-Ce6 preparation. (B) Transmission electron microscopy (TEM) images of ^KK^Ftn protein (negative staining with 1% uranyl acetate). (C) TEM images of MFtn-Ce6 with biomineralization of Fe_3_O_4_ nanoparticles. (D) DLS analysis of ^KK^Ftn, MFtn, and MFtn-Ce6. (E) X-ray photoelectron spectroscopy analysis of MFtn-Ce6. (F) Time-dependent measurement of ^1^O_2_ generation using the DPBF assay. Free Ce6 and MFtn-Ce6 (both contain 4 μg ml^−1^ Ce6) were used in the assay with 25 μg ml^−1^ DPBF. PBS was used as reference with 25 μg ml^−1^ DPBF. The percentage of DPBF remaining was calculated based on the UV absorption of DPBF at different times. (G) Electron spin resonance spectra of ^1^O_2_ trapped by 2,2,6,6-tetramethylpiperidine. Samples containing 4 μg ml^−1^ Ce6 were used in both free Ce6 and MFtn-Ce6.

^KK^Ftn showed a hollow spherical structure on transmission electron microscopy (Fig. 2B). The diameter of the ^KK^Ftn was approximately 12.9 nm and the inner cavity was approximately 8 nm. After biomineralization, the uniformly monodispersed Fe_3_O_4_ core (3 to 4 nm) can be clearly observed (Fig. 2C). Dynamic light scattering (DLS) measurements show that the fusion of 2 lysine residues through a (GS)_3_ linker neglects the influence on the hydrodynamic diameter of Ftn. After biomineralizing the Fe_3_O_4_ core, the hydrodynamic diameter of ^KK^Ftn slightly increased to 15.8 nm, indicating that the Fe_3_O_4_ was generated in the internal cavity of ^KK^Ftn. Additionally, a slight increment of hydrodynamic diameter (1.2 nm) was observed upon the conjugation of Ce6 (Fig. 2D), which can be attributed to the surface modification of the protein. The successful conjugation of Ce6 to MFtn was confirmed by the characteristic absorption peak of Ce6 on the UV–vis spectroscopy of MFtn-Ce6 (Fig. S2). The absorbance at 656 nm indicates that Ce6 was loaded on each MFtn-Ce6 cage. Measurement of the amount of drug loaded on MFtn indicated the high ligation efficiency in the preparation of MFtn-Ce6 (Fig. S1). A drug loading ratio of 2.79 ± 0.109% (w/w) was obtained in the reaction of 100 μg/ml Ce6, corresponding to about 24 Ce6 on each ^KK^Ftn cage (Table S1).

The schematic diagram of the preparation of MFtn-Ce6 is shown in Fig. 2A. To obtain active sites on the surface of Ftn, 2 extra lysine residues were fused with Ftn at the N-terminus (^KK^Ftn) *via* a linker sequence (GS)_3_, which provides active NH_2_ groups for the site-specific anchor of Ce6. The flexible linker can minimize the influence of Ce6 on the function of Ftn. The expression and purification of ^KK^Ftn were performed as described previously with minor modifications [27]. Then, the ferrimagnetic Ftn (MFtn) was synthesized by in vivo biomineralization using the ^KK^Ftn template and Fe^2+^, and the MFtn was purified by centrifugation followed by dialysis [28]. By using inductively coupled plasma–atomic emission spectrometry, iron concentration in ^KK^Ftn was determined, showing approximately 2,000 Fe in each ^KK^Ftn cage. After biomineralization of the magnetic iron oxide core in Ftn, Ce6 was covalently conjugated onto MFtn through the amidation reaction with the amino groups of lysine residues on the MFtn surface using activated carboxyl groups on Ce6, generating MFtn-Ce6. The prepared MFtn-Ce6 was purified by ultrafiltration and stored in Na_2_CO_3_/NaHCO_3_ buffer (pH 8.0) at 4 °C for future use.

X-ray photoelectron spectroscopy was used to analyze the valence of Fe in MFtn-Ce6. The 2 characteristic peaks for Fe 2p_1/2_ (723.9 eV) and Fe 2p_3/2_ (710.4 eV) confirm the presence of Fe(II) and Fe(III) (Fig. 2E), indicating the formation of Fe_3_O_4_ in MFtn (Fig. 2E) [29]. These results confirmed the successful biomineralization of Fe_3_O_4_ in the cavity of Ftn and the modification of Ce6 on the protein surface. Furthermore, the stability of MFtn-Ce6 was assessed by monitoring the changes in particle size in cell culture medium containing 10% fetal bovine serum (FBS). DLS measurement showed that the size of MFtn-Ce6 had a negligible change in 72 h (Fig. S3), suggesting the high stability of MFtn-Ce6, which is essential for biomedical applications. Then, the release behavior of Ce6 in the covalently bound MFtn-Ce6 and the encapsulated MFtn@Ce6 was evaluated. Nearly 20% of Ce6 was released from MFtn@Ce6 during 8 h of incubation (Fig. S4), suggesting the possible leakage of Ce6 during the in vivo circulation. By comparison, nearly no Ce6 was released from MFtn-Ce6 in 48 h, which can be anticipated since the stable covalent binding can prevent the cargo release.

The photodynamic property of MFtn-Ce6 was analyzed by measuring the generation of ^1^O_2_ using the trapping agent 1,3-diphenylisobenzofuran (DPBF). The time-dependent decrease of the absorption of DPBF indicates the effective generation of ^1^O_2_ after irradiation on free Ce6 and MFtn-Ce6 (Fig. 2F and Fig. S5). The rate of the decrease suggests the comparable efficiency of reactive oxygen species (ROS) production of free Ce6 and MFtn-Ce6. Furthermore, electron spin resonance spectroscopy was applied to verify the generation of ^1^O_2_ by using 2,2,6,6-tetramethylpiperidine as a spin trap agent. The characteristic triplet peaks indicated the production of ^1^O_2_ under the irradiation of MFtn-Ce6 (Fig. 2G). These results indicate that the Ce6 maintains its photodynamic property after covalent conjugation to ^KK^Ftn; thus, MFtn-Ce6 is suitable for further PDT applications.

### Photodynamic properties of MFtn-Ce6 in vitro

**Fig. 3.**
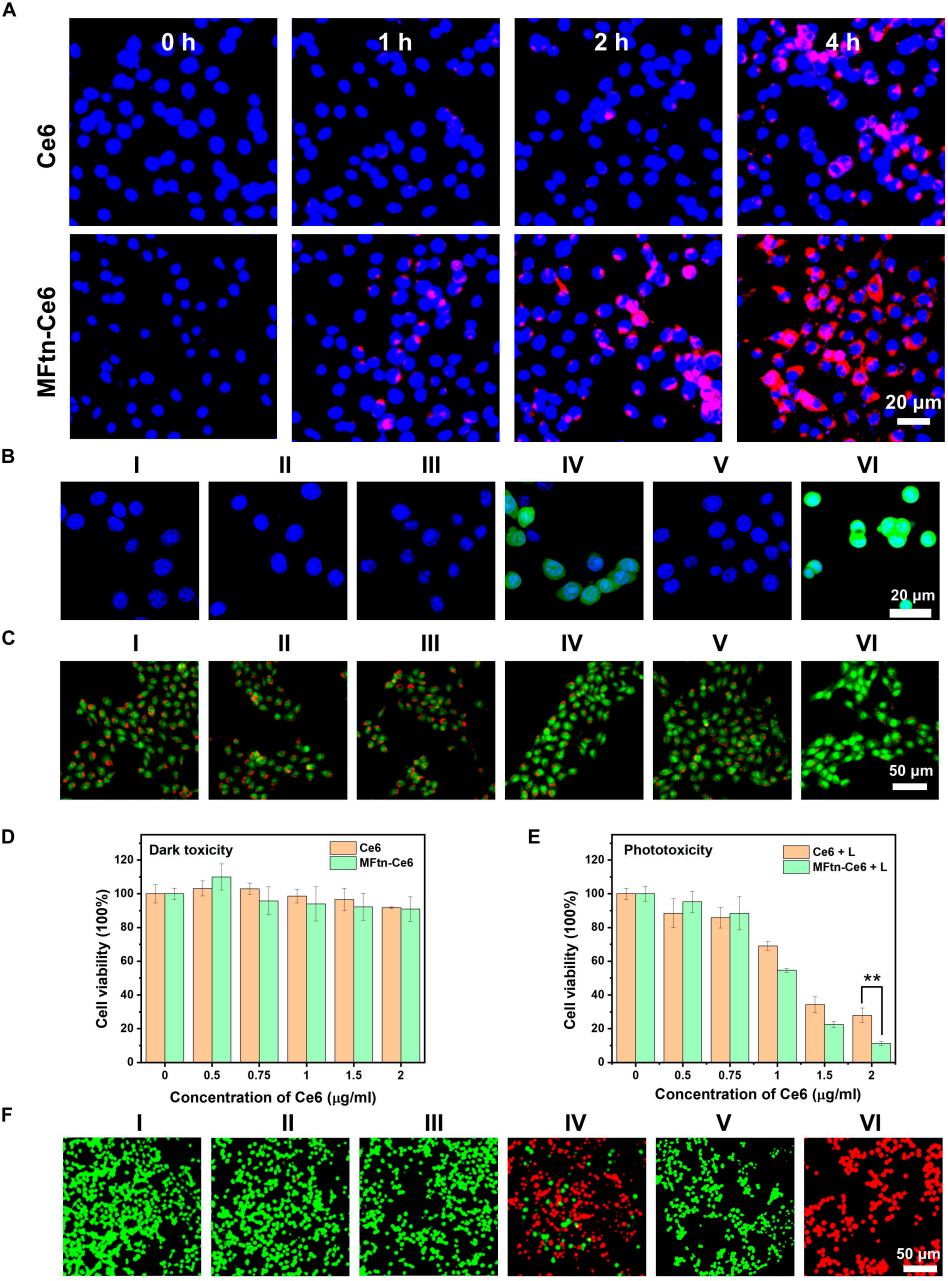
Photodynamic effect of MFtn-Ce6 on tumor cells. (A) Confocal fluorescence microscopic measurement of the cellular uptake of MFtn-Ce6 and Ce6 (red fluorescence for Ce6 and blue for nucleus) after different incubation times. Ce6 (20 μg ml^−1^) was used in both free Ce6 and MFtn-Ce6. (B) ROS generation in 4T1 cells. Ce6 (2 μg ml^−1^) was used in both free Ce6 and MFtn-Ce6 for 4-h incubation. Laser (25 mW cm^−2^, 660 nm, 5 min); DCFH-DA (10 μM); Hoechst 33342 (10 μg ml^−1^). (C) Fluorescence imaging of AO-stained 4T1 cells. Ce6 (2 μg ml^−1^) was used in both free Ce6 and MFtn-Ce6 for 4-h incubation. Laser (25 mW cm^−2^, 660 nm, 5 min); AO (10 μM). (D) Viability of 4T1 cells without laser irradiation after treatment with Ce6 or MFtn-Ce6 in different concentrations. (E) Viability of 4T1 cells with laser irradiation after treatment with Ce6 or MFtn-Ce6 in different concentrations. Laser (25 mW cm^−2^, 660 nm, 5 min). (F) Cell live/dead stain assay. Ce6 (2 μg ml^−1^) was used in both free Ce6 and MFtn-Ce6 and incubated for 4 h (660-nm laser, 25 mW cm^−2^, 5 min). The 4T1 cells were stained with 5 μM FDA (green for live cells) and 10 μM PI (red for dead cells). Group I: PBS; Group II: PBS plus laser; Group III: Ce6; Group IV: Ce6 plus laser; Group V: MFtn-Ce6; and Group VI: MFtn-Ce6 plus laser.

The cellular uptake of MFtn-Ce6 was evaluated, as the efficient cellular internalization and retention of PSs in tumor cells is an effective way for enhancing the PDT efficacy. Confocal FLI showed that, after incubation with MFtn-Ce6 for 1 h, the red fluorescence of Ce6 can be clearly observed in cells, and the number and brightness of cells with red fluorescence increased with incubation time (Fig. 3A and Fig. S6). In comparison, much lower fluorescence was observed on cells incubated with free Ce6 under the same conditions. This result indicates that the Ftn conjugation enhances the cellular uptake of MFtn-Ce6 in comparison to free Ce6. Furthermore, the cellular uptake of free Ce6 and MFtn-Ce6 in tumor cells was measured on cells with different levels of TfR1 expression (TfR1^+^ 4T1 cells and TfR1^−^ 3T3 cells). Both confocal microscopy and flow cytometry results showed that the cellular uptake of MFtn-Ce6 was higher in 4T1 cells than in 3T3 cells (Fig. S7), confirming the good targeting ability and selectivity of MFtn-Ce6 to TfR1^+^ cells.

Notably, the covalent conjugation of Ce6 to Ftn (MFtn-Ce6) exhibits even more efficient cellular uptake over the encapsulation of Ce6 in the cavity of Ftn (MFtn@Ce6) (Fig. S8). This result highlights the advantages of covalent ligation of PS molecules to Ftn. It was well-known that the cellular efflux of small molecular drugs is one of the major causes leading to drug resistance [30,31]. The free Ce6 can be pumped out quickly after releasing from Ftn cavity. The covalent conjugation of Ce6 in Ftn prevents the premature leakage of PSs and, hence, exhibits higher cellular accumulation of Ce6 than the encapsulation of Ce6 in the cavity of Ftn (MFtn@Ce6), which would be beneficial for PDT applications.

Encouraged by the efficient cellular uptake of MFtn-Ce6, the ROS generation in cells was further analyzed using a ROS probe 2,7-dichlorofluorescein diacetate (DCFH-DA). DCFH-DA can be internalized into cells and oxidized by cellular ^1^O_2_, generating bright green fluorescent dichlorofluorescein in cells. Fluorescence measurements indicate that the cells treated with MFtn-Ce6 produced a large amount of ROS upon laser irradiation (660 nm) (Fig. 3B and Fig. S9, green signals). By comparison, free Ce6 showed much less effective ROS generation in cells under the same Ce6 concentration. The strong ROS production by MFtn-Ce6 is consistent with its efficient cellular uptake.

The lysosomal integrity was evaluated since excessive ROS is able to impair lysosomal membranes [32]. Acridine orange (AO) is a dichromatic dye that appears red when it is accumulated in acidic lysosomes. The disruption of lysosomal membrane causes diffusion of AO into the cytoplasm and nucleus and results in the green fluorescence [33]. FLI shows that laser irradiation clearly reduced the red fluorescence in the cells treated with free Ce6; more significantly, the red fluorescence was almost extinguished in cells treated with MFtn-Ce6 and only green signals can be detected (Fig. 3C). This result confirms that the ROS generated *via* PDT led to extensive disruption of lysosomal membrane.

Methyl thiazolyl tetrazolium (MTT) assay was performed to analyze the effect of MFtn-Ce6 on cell proliferation. The treatment of free Ce6 and MFtn-Ce6 without laser irradiation did not cause apparent inhibition of cell growth (Fig. 3D); nevertheless, the cell proliferation was greatly decreased after laser exposure at 660 nm. Consistent with ROS generation and lysosomal membrane disruption, MFtn-Ce6 showed a more pronounced effect than free Ce6 on cell proliferation. With 2 μg ml^−1^ Ce6, the viability of cells was reduced to approximately 11% in the MFtn-Ce6 group and 30% in free Ce6 (Fig. 3E). To visualize the effect more intuitively, a live/dead assay was performed. As expected, without laser irradiation, free Ce6 or MFtn-Ce6 did not affect the cell viability. After laser irradiation, almost all cancer cells treated with MFtn-Ce6 were dead (red fluorescence). By comparison, a small amount of living cells existed in the cells treated with free Ce6 (green fluorescence) (Fig. 3F and Fig. S10). Cell apoptosis assessment confirmed that the free Ce6 and MFtn-Ce6 caused little apoptosis of cells, while the laser exposure shot up the apoptotic level to 57.8% and 92.1%, respectively (Fig. S11). These results confirm that the MFtn-Ce6 nanoplatform exhibits a more powerful PDT effect and phototoxicity than the free Ce6 on cancer cells.

### Dual-modality imaging of material biodistribution in vivo

**Fig. 4.**
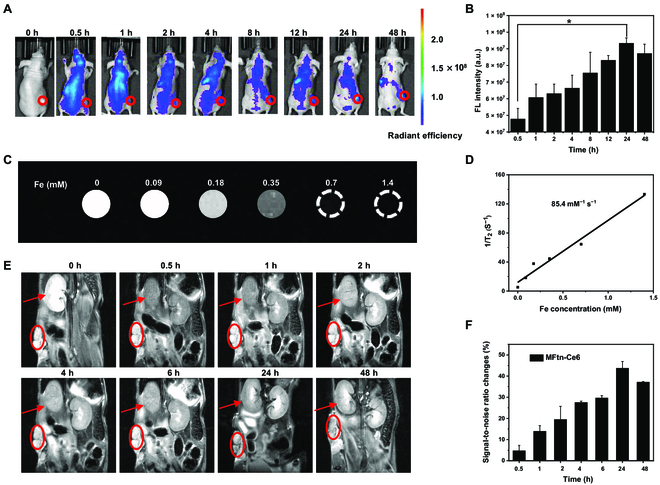
The dual-modality imaging of MFtn-Ce6 in vitro and in vivo. (A) In vivo FLI of tumor-bearing mice treated with MFtn-Ce6. The interval time of FLI after MFtn-Ce6 treatment is labeled in the figure. The red circles indicate tumor sites (200 μl of 200 μg ml^−1^ Ce6 was used in both free Ce6 and MFtn-Ce6). (B) The quantification of fluorescence intensity at the tumor site at each time point in panel (A). (C) The *T*_2_-weighted maps of MFtn-Ce6 at different conditions. (D) The transverse relaxation rate (1/*T*_2_) of MFtn-Ce6 on the basis of Fe concentrations. (E) In vivo *T*_2_-weighted MR images of the tumor (red oval) and kidney (red arrow) after injecting with MFtn-Ce6. (F) The corresponding signal-to-noise ratio changes (∆SNR) of panel (E).

Inspired by the cancer cell targeting and PDT effect on cell-based assays, the in vivo biodistribution and metabolism of MFtn-Ce6 were investigated on BALB/c mice bearing 4T1 engraftment tumors. The near-infrared fluorescence property of Ce6 allows direct assessment of the in vivo fate of MFtn-Ce6 using FLI. In vivo FLI showed that, after intravenous administration of MFtn-Ce6, fluorescence signal can soon be observed throughout the body (Fig. 4A). While the overall fluorescence decreased rather quickly, the signal gradually increased at the tumor site and can be distinctly noticed from 4 h after injection. The intensity became stronger and reached a peak at 24 h. It is noteworthy that the fluorescence levels remained high even at 48 h post-injection (Fig. 4B). The ex vivo FLI (recorded on mice sacrificed at 48 h post-injection) confirmed the enrichment of MFtn-Ce6 in tumor even after 48 h (Fig. S12). Although the high accumulation in liver can be observed due to the inevitable capture of nanoparticles by the metabolic organs [34], there was negligible accumulation of Ce6 in other organs, which indicated that MFtn-Ce6 can be gradually cleared over time while enriching in tumor sites. Compared with MFtn-Ce6, free Ce6 was detected in the tumor region starting at 0.5 h post-injection; however, it was quickly eliminated in 8 h (Fig. S13). This result reveals that the MFtn-Ce6 nanoplatform allows Ce6 to lodge in tumor tissues for a long time and less residual in other sites. The cellular internalization-induced tumor accumulation provides a prolonged time window for PDT using MFtn-Ce6.

The magnetic Fe_3_O_4_ nanoparticles biomineralized in the inner cavity of Ftn confer MRI feature to the nanoplatform. The contrast enhancement property can be directly observed by the Fe-concentration-dependent darkness increase of MFtn-Ce6 in tubes (Fig. 4C). Accordingly, the transverse relaxation of MFtn-Ce6 was calculated, giving a *T*_2_ relaxation rate of 85.4 mM^−1^ s^−1^ (Fig. 4D). This value is higher than that of the commercial superparamagnetic iron oxide nanoparticles (50.2 mM^−1^ s^−1^) [35]. The result demonstrates that MFtn-Ce6 exhibits remarkable transverse relaxation property. Hence, MRI of MFtn-Ce6 was measured on a 9.4-T MR scanner. After injection via the tail vein, the darkness of the tumor area increased over time (Fig. 4E), suggesting the accumulation of MFtn-Ce6 in tumor. The gradual darkening and recovery of the kidney area indicates that the metabolism of MFtn-Ce6 in the kidney is relatively fast (Fig. S14). To determine the signal-to-noise ratio (SNR), the mean signal in the region of interest and the standard deviation from pixels located in the background were measured. Correspondingly, the alteration of signal-to-noise ratio (ΔSNR) in the tumor region indicates that the most effective MRI [36], which corresponds to the accumulation of MFtn-Ce6 in tumor, can be reached at 24 h after administration (Fig. 4F). The reduced darkness after 24 h suggests the gradual clearance of MFtn-Ce6. The result is consistent with the in vivo FLI that the MFtn-Ce6 can be enriched in tumor in 24 h. The consistency of 2 imaging results, by measuring the covalent decoration of Ce6 on the Ftn surface and encapsulating Fe inside the cavity, suggests the high stability of MFtn-Ce6 in vivo for at least 48 h.

### Efficacy and safety evaluation of PDT in vivo

**Fig. 5.**
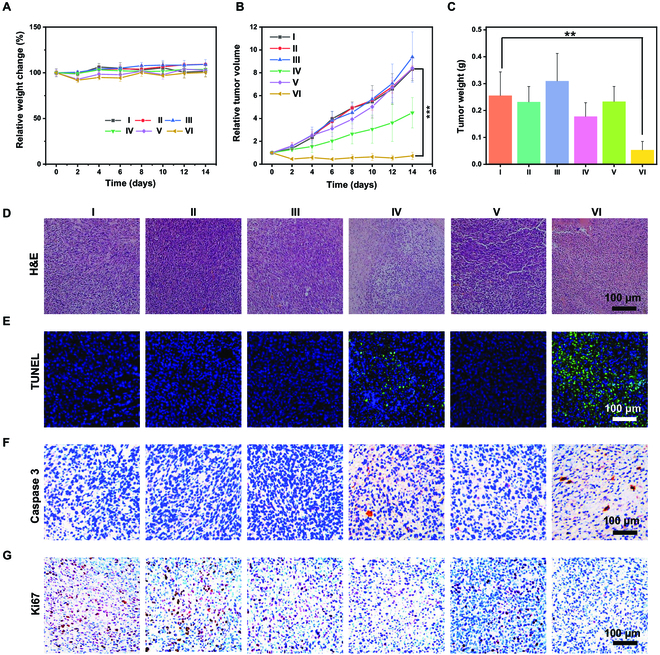
In vivo antitumor effect of MFtn-Ce6. (A) Body weight and (B) tumor volume changes during treatment. (C) Tumor weight at the end of the experiment. (D) H&E-stained images of tumors. (E) TUNEL-stained images of tumors (blue fluorescence: Hoechst; green fluorescence: TUNEL). (F) Caspase-3 immunostaining of the tumor tissues. (G) Ki67 immunostaining of the tumor tissues (scale bar = 100 μm). Mice received different treatments: I (PBS), II (PBS plus laser), III (Ce6), IV (Ce6 plus laser), V (MFtn-Ce6), and VI (MFtn-Ce6 plus laser). Dosage: 200 μl of 0.2 mg ml^−1^ Ce6 and 0.2 mg ml^−1^ Ce6 with MFtn-Ce6; near-infrared (NIR) irradiation: 25 mW cm^−2^ 660-nm laser for 10 min (*n* = 4; *P < 0.05, **P < 0.01, ***P < 0.001 [*t* test]).

The effect of targeted PDT with MFtn-Ce6 has been evaluated on 4T1 Balb/c mouse models. Mice were randomly assigned to 6 groups when the tumor volume reached approximately 70 mm^3^. Groups I to VI were treated with PBS, PBS plus laser, Ce6, Ce6 plus laser, MFtn-Ce6, and MFtn-Ce6 plus laser, respectively. The above treatments were administered 3 times every other day through tail vein injection. Then, the tumor regions of the II, IV, and VI groups were irradiated with a 660-nm laser at 25 mW cm^−2^ for 10 min after 8 h injection of free Ce6 or MFtn-Ce6. The tumor volume and the body weight of the mice were measured every other day during treatment. The result shows that the body weight of the mice remained basically stable in all groups (Fig. 5A), suggesting their low toxic side effect. Compared with group I (PBS), groups II (PBS plus laser), III (Ce6), and V (MFtn-Ce6) exhibited a negligible effect on tumor inhibition. The laser irradiation clearly suppressed tumor growth in the mice treated with Ce6. Remarkably, the tumors were nearly abolished with the combination of MFtn-Ce6 and laser irradiation (group VI); the tumors were even smaller than before treatment at just approximately 70% of the original volume (Fig. 5B). Mice were euthanized after 14 days of treatment and tumors were taken for further examination (Fig. 5C). The tumor weight results were in accordance with the tumor growth curve, and representative tumor images further confirm these results (Figs. S15 and S16). It can be speculated that the targeting and accumulation of MFtn-Ce6 to tumor cells promote the PDT effect.

After the treatment, tumors were strained with hematoxylin and eosin (H&E) staining for histopathological analysis. No obvious histological damages were detected in major organs from all the treatment groups, indicating no notable toxicity of these agents (Fig. S17). However, the tumor tissues of mice treated with free Ce6 plus laser irradiation showed obvious damage compared with the control group (Fig. 5D), and this phenomenon was strongly enhanced in group VI, implying the excellent PDT effect of MFtn-Ce6. Moreover, the therapeutic efficacy of different groups in vivo was also investigated through the terminal deoxynucleotidyl transferase-mediated deoxyuridine triphosphate nick end labeling (TUNEL) staining and caspase-3 immunostaining. The TUNEL analysis showed an abundance of TUNEL-positive green signals in group VI, which revealed that the MFtn-Ce6 caused intracellular oxidative stress and contributed to cell apoptosis (Fig. 5E). To further verify the occurrence of apoptotic events induced by the PDT effect of MFtn-Ce6, the expression level of caspase-3 in tumors was analyzed. It was obvious that the caspase-3 expression was considerably increased after treatment with MFtn-Ce6 plus laser irradiation (Fig. 5F), confirming that the tumor cell death was associated with PDT-mediated apoptosis. The cell proliferation was also analyzed by Ki67 staining. A notable reduction in Ki67-positive cells can be found in group VI, indicating that the MFtn-Ce6 plus laser significantly reduced the proliferation of tumor cells (Fig. 5G).

## Discussion

PDT has been extensively studied as a noninvasive treatment for tumors. To enhance PDT efficacy, nanocarriers for tumor-specific delivery of PSs have been widely used to improve the solubility, biodistribution, and bioavailability of PSs. However, the PS concentration in tumor sites is often lower than desired therapeutic levels due to the unavoidable leakage from carriers. The free PSs could be pumped out quickly after release from the nanocarriers. The covalent conjugation of PSs in protein carriers would prevent the premature leakage of PSs. In this work, a ferrimagnetic Ftn was constructed for specific site ligation of Ce6 on the surface of the protein to obtain a theranostic nanoplatform (MFtn-Ce6). The covalent linking Ce6 is more stable compared with the simple encapsulation ones inside the cavity of Ftn, which minimized the premature leakage of PS, and prolongs the in vivo circulation and increases the retention of Ce6 in tumor sites.

It was worth noting that, to obtain active sites on the surface of Ftn, 2 extra lysine residues were fused with Ftn at the N-terminus (^KK^Ftn) via a linker sequence (GS)_3_, which provides active NH_2_ groups for the site-specific anchor of Ce6. The flexible linker can minimize the influence of Ce6 on the function of Ftn. These 2 residues are more reactive than other lysine residues since the flexible linker makes the residues more accessible on the surface of Ftn. This approach, with site-specific modification and flexible linker, minimizes alterations of the structure and functions of Ftn, and avoids premature release of the loading cargoes. In addition, this dual-modality imaging allows cross-checking biodistribution of MFtn-Ce6 in vivo. Moreover, the consistent imaging results of 2 methods imply the high stability of MFtn-Ce6 during circulation, as the FLI agent Ce6 and the MRI agent iron oxide core were located at different parts of the nanoplatform. In addition, the dual-modality imaging combines the advantages of 2 methods based on their inherent properties, including the high sensitivity and low cost of FLI and the high spatial resolution and deep penetration of MRI [37–39].

In conclusion, a tumor-targeted drug delivery nanoplatform has been designed using engineered Ftn with covalent conjugation of PS (Ce6) for minimizing the premature drug release and enhancing drug distribution in tumor. The fusion of additional lysine residue and flexible spacer enables site-specific modification of Ftn without perturbing the protein functions. The engineered Ftn can efficiently encapsulate magnetic iron oxide nanoparticles *via* biomineralization and covalently conjugate the PS Ce6 on its surface to generate a theranostic platform MFtn-Ce6 for tumor diagnosis and PDT. This nanoplatform allows dual-modality imaging with fluorescence and MRI, which is capable of monitoring the distribution of therapeutic agents in real time with high spatial resolution of MRI and high sensitivity of fluorescence. The time-dependent in vivo imaging enables the optimization of the time window for PDT treatment. The result shows that MFtn-Ce6 is gradually enriched into tumors in 24 h and maintains good retention in tumor for at least 48 h. Meanwhile, MFtn-Ce6 can be cleared rather quickly from other organs. By comparison, MFtn-Ce6 demonstrated higher and prolonged tumor accumulation than free Ce6. The MFtn-Ce6 allows efficacious PDT treatment, resulting in significant tumor depletion. This work established a general approach for generating theranostic nanoplatform by using engineered Ftn with drug encapsulation in the protein cavity and covalent conjugation of therapeutic agents on the protein surface.

## Materials and Methods

### Synthesis of MFtn

The in situ mineralization was performed by slowly pumping (NH_4_)_2_Fe(SO_4_)_2_⋅6H_2_O (12.5 mM at 158 μl/min) and H_2_O_2_ (4.17 mM) into ^KK^Ftn solution (1 mg ml^−1^, containing 100 mM NaCl, at pH 8.5, 65 °C). After mineralization, sodium citrate solution was added to remove free iron ions. Then, MFtn was obtained by removing small molecules through dialysis and concentrated by centrifugation.

### Synthesis of MFtn-Ce6

Ce6 (2 mg), N-hydroxysuccinimide (NHS, 4.6 mg), and 1-ethyl-3-(3΄-dimethylaminopropyl)carbodiimide (EDC, 7 mg) were dispersed in dimethyl sulfoxide (DMSO) and stirred for 1 h. Then, 10 ml of 2 mg ml^−1^ MFtn was added and incubated in Na_2_CO_3_/NaHCO_3_ buffer (pH 8.0) overnight. MFtn-Ce6 was obtained by removing aggregates via centrifugation and removing unreacted small molecules through ultrafiltration.

### Cellular assays in vitro

All cellular assays were performed in the following procedure unless specified. 4T1 cells (1 × 10^5^) were seeded in 6-well plates, incubated overnight before adding the given amount of Ce6 or MFtn-Ce6 (equivalent of Ce6), and incubated for another 4 h before analyses. Laser irradiation was applied with 25 mW/cm^2^ at 660 nm for 5 min.

Cellular uptake was analyzed by the treatment of Ce6 or MFtn-Ce6 (20 μg ml^−1^) for different times. Then, the cells were washed and imaged by a fluorescence microscope.

To verify the tumor targeting property of MFtn-Ce6, 1 × 10^5^ 4T1 cells and NIH-3T3 cells were treated with Ce6 or MFtn-Ce6 (20 μg ml^−1^) and incubated for 4 h at 4 °C. Then, the cells were washed and imaged by confocal microscopy.

Cell viability assay was performed on 5 × 10^3^ 4T1 cells in 96-well plates with treatment of Ce6 or MFtn-Ce6 (0 to 2 μg ml^−1^). After incubating for another 24 h, an MTT solution was added and the absorbance at 490 nm was measured by a Bio-Rad 680 microplate reader.

For cell live/dead assay, cells were treated with Ce6 (2 μg ml^−1^) or MFtn-Ce6 (equivalent to 2 μg ml^−1^ Ce6). After laser irradiation, cells were incubated for another 12 h, and then the cells were washed and stained with fluorescein diacetate (FDA, 10 μM) and propidium iodide (PI, 20 μM) before recording FLI.

Cellular ROS levels were measured by the treatment of Ce6 or MFtn-Ce6 (2 μg ml^−1^) for 4 h. Cells were washed and analyzed with an ROS Assay Kit (Beyotime Biotechnology) and Hoechst 33342 (10 μg ml^−1^) with a fluorescence microscope.

Apoptosis assay was performed on cells treated with Ce6 or MFtn-Ce6 (2 μg ml^−1^) for 4 h. After laser irradiation, cells were incubated for another 12 h and then the cells were washed and treated with Annexin V-FITC and PI and analyzed by flow cytometry.

Lysosomal damage was evaluated on cells treated with Ce6 or MFtn-Ce6 (2 μg ml^−1^) for 4 h. The irradiation group was irradiated with a 25 mW/cm^2^ 660-nm laser for 5 min. After laser irradiation, cells were incubated for another 12 h, washed and stained with AO (10 μM), and imaged by fluorescence microscopy.

### In vivo FLI and MRI

MFtn-Ce6 and free Ce6 (200 μl of 200 μg ml^−1^ Ce6) were injected into mice via the tail vein. The signals were monitored at different time points on an IVIS imaging system and a 9.4-T MR scanner.

### In vivo PDT

4T1 tumor-bearing mice were randomly divided into 6 groups with the following treatments: PBS, PBS plus laser, Ce6, Ce6 plus laser, MFtn-Ce6, and MFtn-Ce6 plus laser. Two hundred microliters of 200 μg ml^−1^ Ce6 or MFtn-Ce6 in equivalent Ce6 was used. PBS (200 μl) was used as a control. At 8 h after injection, laser irradiation was applied on laser groups (25 mW/cm^2^, 660 nm, 10 min). The administrations were performed every other day at the beginning of treatment and repeated 3 times. The body weight and tumor volume were monitored every 2 days after treatment. At the end of the 14-day treatment, mice were euthanized and major organs were collected for immunohistochemical/immunofluorescence analysis.

## Data Availability

All data are available within the article and Supplementary Materials, or available from the authors upon request.

## Supplementary Materials

Supplementary 1 Supplementary Materials and Methods Fig. S1. Encapsulation efficiency of Ce6 on MFtn-Ce6. Fig. S2. The UV–vis absorbance spectra of MFtn and MFtn-Ce6. Fig. S3. The stability of MFtn-Ce6 in RPMI with 10% FBS. Fig. S4. The release behavior of Ce6 in MFtn-Ce6 and MFtn@Ce6. Fig. S5. ^1^O_2_ generation efficiency measured by DPBF assay at different time points. Fig. S6. The cellular uptake of Ce6 and MFtn-Ce6. Fig. S7. The cell uptake of MFtn-Ce6 and Ce6 in 3T3 and 4T1 cells. Fig. S8. The cellular uptake of MFtn-Ce6 and MFtn@Ce6 at 12 h. Fig. S9. ROS generation in 4T1 cells. Fig. S10. Live/dead cell assay. Fig. S11. Flow cytometric analysis of cell apoptosis. Fig. S12. Ex vivo fluorescence images of the major organs and tumors. Fig. S13. Real-time bio-distribution images of free Ce6 after injection. Fig. S14. Signal-to-noise ratio (ΔSNR) changes of in vivo *T*_2_-weighted MR images after injection of MFtn-Ce6. Fig. S15. The photographs of xenograft tumors excised from mice. Fig. S16. Photographs of mice after 14-day treatment. Fig. S17. Pathological H&E-stained tissue sections. Table S1. The summary of encapsulation efficiency and drug-loading rate of the MFtn-Ce6 platform.
