# Supplementary material for: A Leaking-Proof Theranostic Nanoplatform for Tumor-Targeted and Dual-Modality Imaging-Guided Photodynamic Therapy
Source: BME Front. 2023 Mar 30;4:0015. doi: 10.34133/bmef.0015 (PMC10085250; doi:10.34133/bmef.0015)
Supplement: Supplementary 1 — Supplementary Materials and Methods Fig. S1. Encapsulation efficiency of Ce6 on MFtn-Ce6. Fig. S2. The UV–vis absorbance spectra of MFtn and MFtn-Ce6. Fig. S3. The stability of MFtn-Ce6 in RPMI with 10% FBS. Fig. S4. The release behavior of Ce6 in MFtn-Ce6 and MFtn@Ce6. Fig. S5. 1O2 generation efficiency measured by DPBF assay at different time points. Fig. S6. The cellular uptake of Ce6 and MFtn-Ce6. Fig. S7. The cell uptake of MFtn-Ce6 and Ce6 in 3T3 and 4T1 cells. Fig. S8. The cellular uptake of MFtn-Ce6 and MFtn@Ce6 at 12 h. Fig. S9. ROS generation in 4T1 cells. Fig. S10. Live/dead cell assay. Fig. S11. Flow cytometric analysis of cell apoptosis. Fig. S12. Ex vivo fluorescence images of the major organs and tumors. Fig. S13. Real-time bio-distribution images of free Ce6 after injection. Fig. S14. Signal-to-noise ratio (ΔSNR) changes of in vivo T2-weighted MR images after injection of MFtn-Ce6. Fig. S15. The photographs of xenograft tumors excised from mice. Fig. S16. Photographs of mice after 14-day treatment. Fig. S17. Pathological H&E-stained tissue sections. Table S1. The summary of encapsulation efficiency and drug-loading rate of the MFtn-Ce6 platform. [file bmef.0015.f1.pdf]

Supporting Information

A Leaking-proof Theranostic Nanoplatform for Tumor-targeted and Dual-modality Imaging-guided Photodynamic Therapy

Duo Jin[1], Yang Zhu[1], Manman Liu[1], Wenxin Yu[1], Jiaji Yu[1], Xinwei Zheng[2], Lulu Wang[2], Yun Wu[2], Kaiju Wei[3], Junjie Cheng[1*], and Yangzhong Liu[1*]

[1]Department of Chemistry, University of Science and Technology of China. Hefei 230001, China.

[2]High Magnetic Field Laboratory, Chinese Academy of Sciences. Hefei 230031, China

[3]Nano Science and technology Institute, Suzhou Institute for Advanced Study, University of Science and Technology of China, Suzhou, 215123, China

Experimental Section

Material

Ammonium iron (II) sulfate hexahydrate ($(NH_4)_2Fe(SO_4)_2$ $6H_2O$), sodium hydroxide (NaOH) and hydrogen peroxide (H_2O_2) were supplied by Sinopharm Chemical Reagent Co., Ltd (Shanghai, China). chlorin e6 (Ce6) were supplied from Shanghai yuanye Bio-Technology Co., Ltd. Roswell Park Memorial Institute (RPMI 1640), fetal bovine serum (FBS), penicillin/streptomycin and Trypsin solution were purchased from Hyclone (Logan, USA). DNase I deoxyribonuclease I (yuanye Bio-Technology Co., Ltd.). Reactive Oxygen Species Assay Kit was purchased from Beyotime Biotechnology. 3-(4,5-dimethylthiazol-2-yl)-2,5-diphenyltetrazolium bromide (MTT) was purchased from Sangon Biotech Co., Ltd. (Shanghai, China). BisBenzimide H 33342 trihydrochloride (Hoechst 33342), 1,3-Diphenylisobenzofuran (DPBF), and 2',7'-dichlorofluorescin diacetate (DCFH-DA), Acridine Orange (AO), fluorescein diacetate (FDA), propidium iodide (PI) were purchased from Beyotime Biological Co., LTD. (Shanghai, China). Other reagents were purchased from Sigma-Aldrich (St. Louis, USA) or Sinophenol Chemical (Shanghai, China). All of these chemicals were used without further purification. Deionized (DI) water is obtained through the Mili-Q Water Purification system (Millibo, Billrica, Massachusetts)

Protein expression and purification

According to methods reported in the literature[1], expression and purification of ^{KK}Ftn which two additional lysine residues were introduced to the N-terminus of ferritin. Briefly, the ^{KK}Ftn clone was expressed in and purified from E. coli. Then the cells were harvested by centrifugation, and subsequently resuspended in 40 mL of resuspended buffer (50 mM Tris, 150 mM NaCl, pH 8.0). The cells were sonicated on ice and the cell lysis was centrifuged to remove E. coli debris. The supernatant was heated at 65 °C for 15 min, during which many of the impure proteins were precipitated and removed by centrifugation. Then solid $(NH_4)_2SO_4$ was added to the obtained supernatant until its concentration was 60%, and the solution was stirred overnight at 4 °C. After centrifuging and removing the supernatant, the protein in the precipitation was resuspended in the buffer of 50 mM Tris, 150 mM NaCl, and pH 8.0. DNA

removal from ^{KK}Ftn using DNAse I. The protein was further purified by size exclusion chromatography (SEC) (GE healthcare Life Sciences, Pittsburgh, PA, USA). The purified ^{KK}Ftn protein were further characterized by UV/Vis spectroscopy, dynamic light scattering (DLS: Brookhaven, 90Plus particle size analyzer) and transmission electron microscopy (TEM: LEO 912AB).

Characterization of MFtn-Ce6

The transmission electron microscopy (TEM) images of MFtn-Ce6 were acquired by a Hitachi-7700 Transmission Electron Microscope. The hydration diameter was conducted through the dynamic light scatterer (Brook Haven) measurements. UV-visible absorption spectroscopy (8453, Agilent) was used for the determination of UV separable absorption. The UV-visible absorption spectroscopy verifies that the photosensitizer was successfully covalently bound to the protein surface. X-ray photoelectron spectroscopy (XPS) studied the valence states of element Fe. Fluorescence imaging by using confocal microscopy (ZEISS710, Carl Zeiss AG) and fluorescence microscope (IX71, Olympus). Metal content analysis by using inductively coupled plasma mass spectrometer (ICP-MS, PlasmaQuad 3, Thermo Elemental). The singlet oxygen was detected by diphenylisobenzofuran (DPBF). The DPBF can capture $^1O_2$ that generated by Ce6 with laser irradiation. The Ce6 (with final concentration of 4 μg mL^{-1}) and MFtn-Ce6 (equivalent concentration of Ce6 with 4 μg mL^{-1}) added 25 μg mL^{-1} DPBF with laser irradiation. Then, the absorbance of samples at 436 nm was measured with a UV-Vis spectrophotometer at different time points. The retained DPBF (%) means the percentage of DPBF remaining, because the UV absorption of DPBF decreased with time. The cell apoptosis and necrosis were monitored by flow cytometer (CytoFLEX, Beckman). Fluorescence imaging using in vivo NIR fluorescence imaging analysis using the IVIS Imaging System. The T_2 relaxation times of progressive concentration of the MFtn-Ce6 were measured with a 9.4T MR scanner.

Drug loading and release

The activated Ce6 (20 - 100 μg mL^{-1}) was added to 2.5 mg mL^{-1} MFtn solution. The UV/Vis

spectroscopy was used to determine the concentration of Ce6 encapsulated on MFtn (C_e). The encapsulation efficiency (EE) was determined by (C_e/C_{total}) × 100%. For measuring Ce6 release, MFtn-Ce6 and MFtn@Ce6 (equivalent 50 μg mL^{-1} Ce6) was dispersed in phosphate buffer (pH 7.4 with 0.5% Tween 80). 2 mL of the MFtn-Ce6 and MFtn@Ce6 were placed in a dialysis bag (MWCO 3k) and dialyzed with 20 mL buffer. The Ce6 in dialysis solution was collected at desirable time points for analysis.

Animals and study approval

Twenty-four female rats (16-20 g, mean 18 g; Balb/c rats from Shanghai Slake Experimental Animal Co., Ltd.). All live animal experiments were conducted according to the Institutional Animal Care and Use Committee of the Animal Experiment Center of University of Science and technology of China (Hefei, China) and the Regulations for the Administration of Affairs Concerning Experimental Animals.

Statistical analysis

All quantitative data are expressed as the mean ± standard deviation (SD). Differences between the two groups were inspected using the Student's two-tailed t-test, and a comparison of multiple groups was performed using one way analysis of variance (ANOVA). The results were considered significant at $*P < 0.05$, $**P < 0.01$, $***P < 0.001$, $****P < 0.0001$, $n \geq 3$.

Supplementary Figures

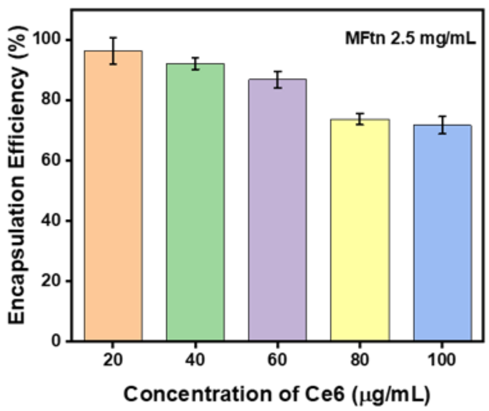

Figure S1. Encapsulation efficiency of Ce6 on MFtn-Ce6. Ce6 (20-100 μg/mL) was added to 2.5 mg/mL MFtn solution. n = 3.

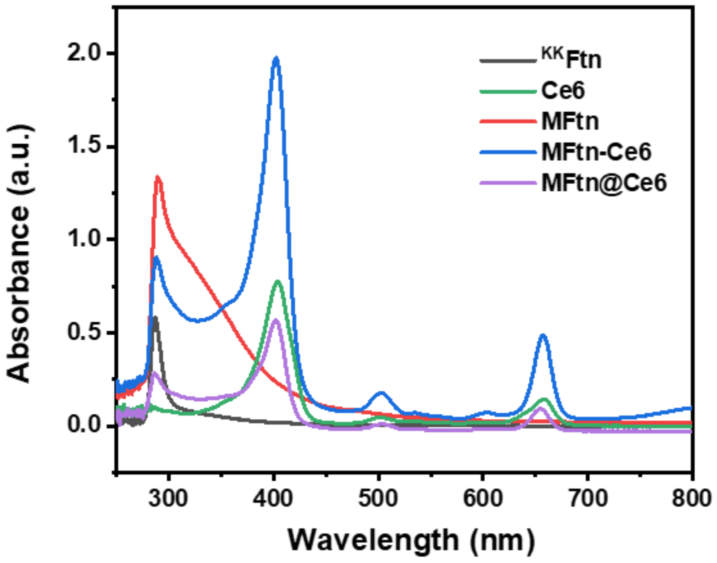

Figure S2. The UV-vis absorbance spectra of ^{KK}Ftn, Ce6, MFtn, MFtn-Ce6 andMFtn@Ce6.

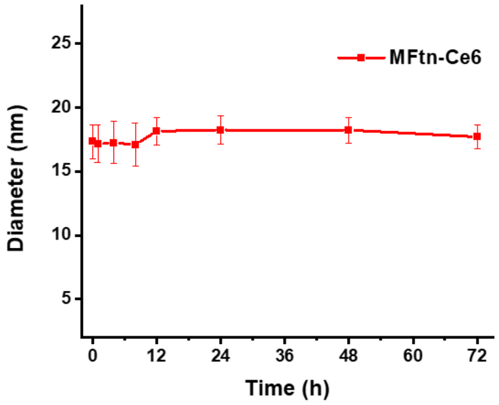

Figure S3. The stability of MFtn-Ce6 in RPMI with 10% FBS. Particle sizes were measured by DLS with different incubation times.

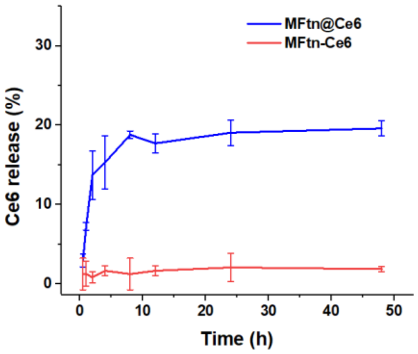

Figure S4. The release behavior of Ce6 in MFtn-Ce6 and MFtn@Ce6 at phosphate buffer (pH 7.4 with 0.5% Tween 80) during 48 h. The concentration of Ce6 in MFtn-Ce6 and MFtn@Ce6 were 50 μg/mL. (n=3)

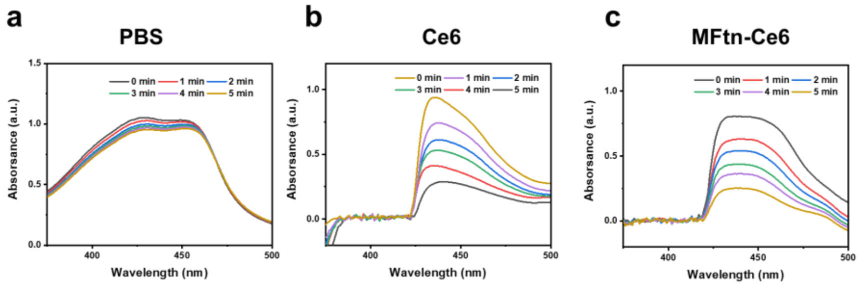

Figure S5. The efficiency of $^{1}O_2$ production was evaluated by the DPBF experiment. The concentration of Ce6 was 4 μg mL^{-1} in both free Ce6 and MFtn-Ce6. 25 μg mL^{-1} DPBF and 660 nm laser 25 mW cm^{-2}.

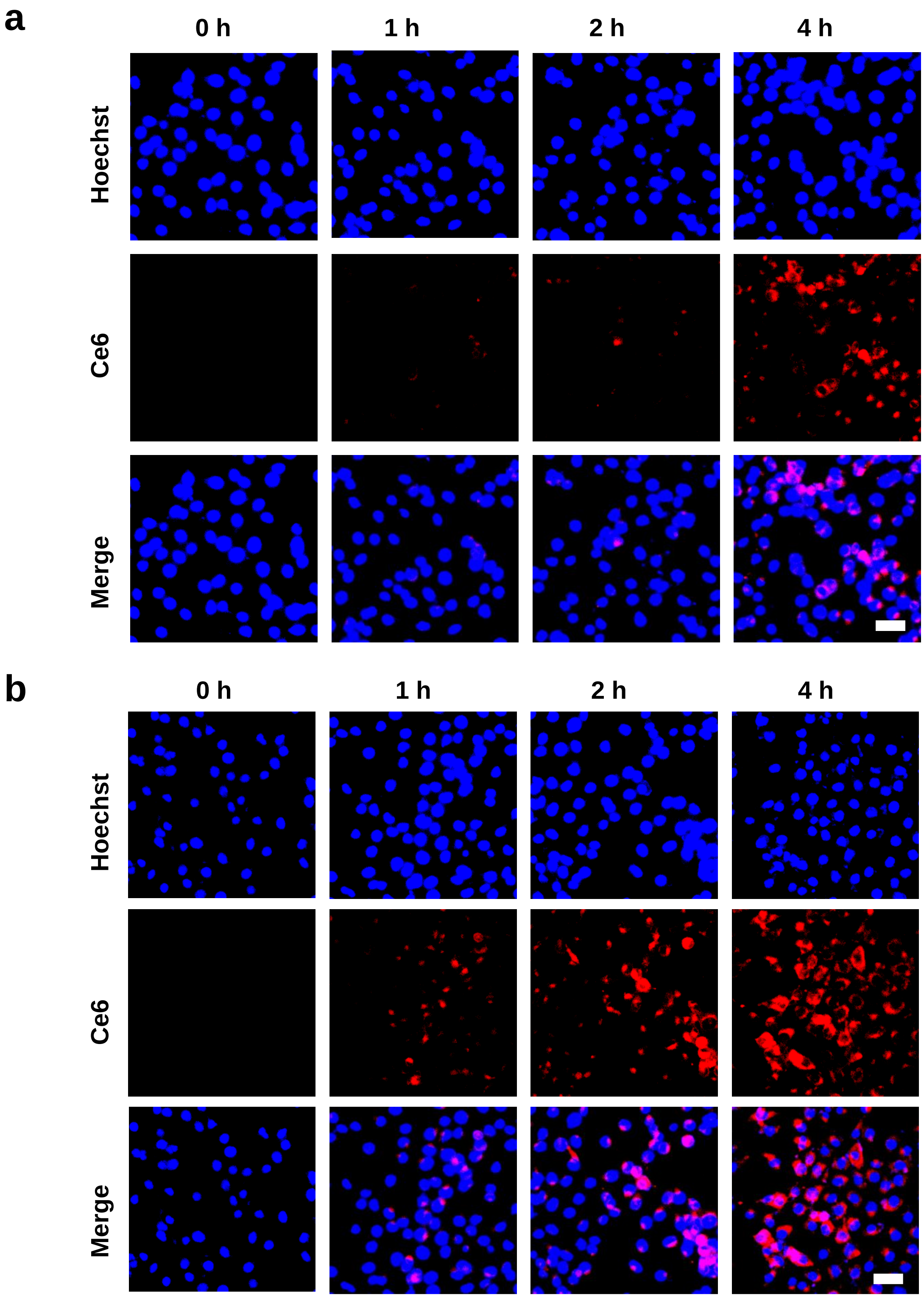

Figure S6. The cellular uptake of MFtn-Ce6 and Ce6. (a) Ce6 and (b) MFtn-Ce6. 20 μg mL^{-1} Ce6, and MFtn-Ce6 equivalent concentration of Ce6 with 20 μg mL^{-1}. Scale bar: 20 μm.

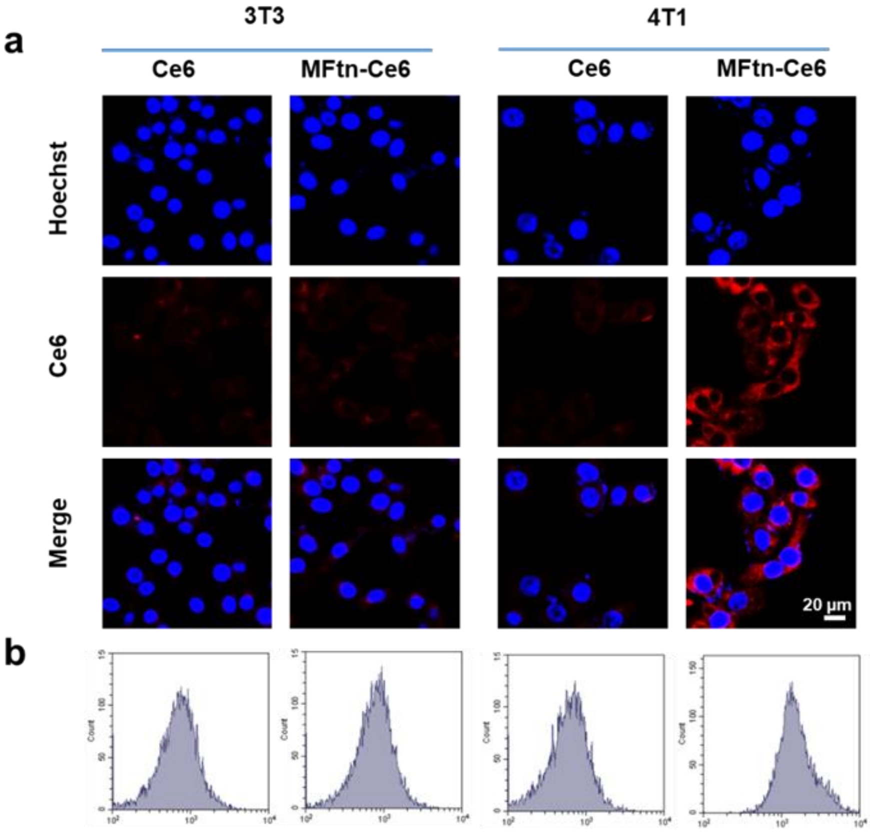

Figure S7. The cell uptake of MFtn-Ce6 and Ce6 in 3T3 and 4T1 cells. (a) confocal microscopy. (b) and flow cytometry. Image depicts merged images of Ce6 (red) and nuclear Hoechst staining (blue). Cells were incubated with 20 μg mL^{-1} Ce6 or equivalent of Ce6 in MFtn-Ce6 for 4 h at 4 ^{o}C.

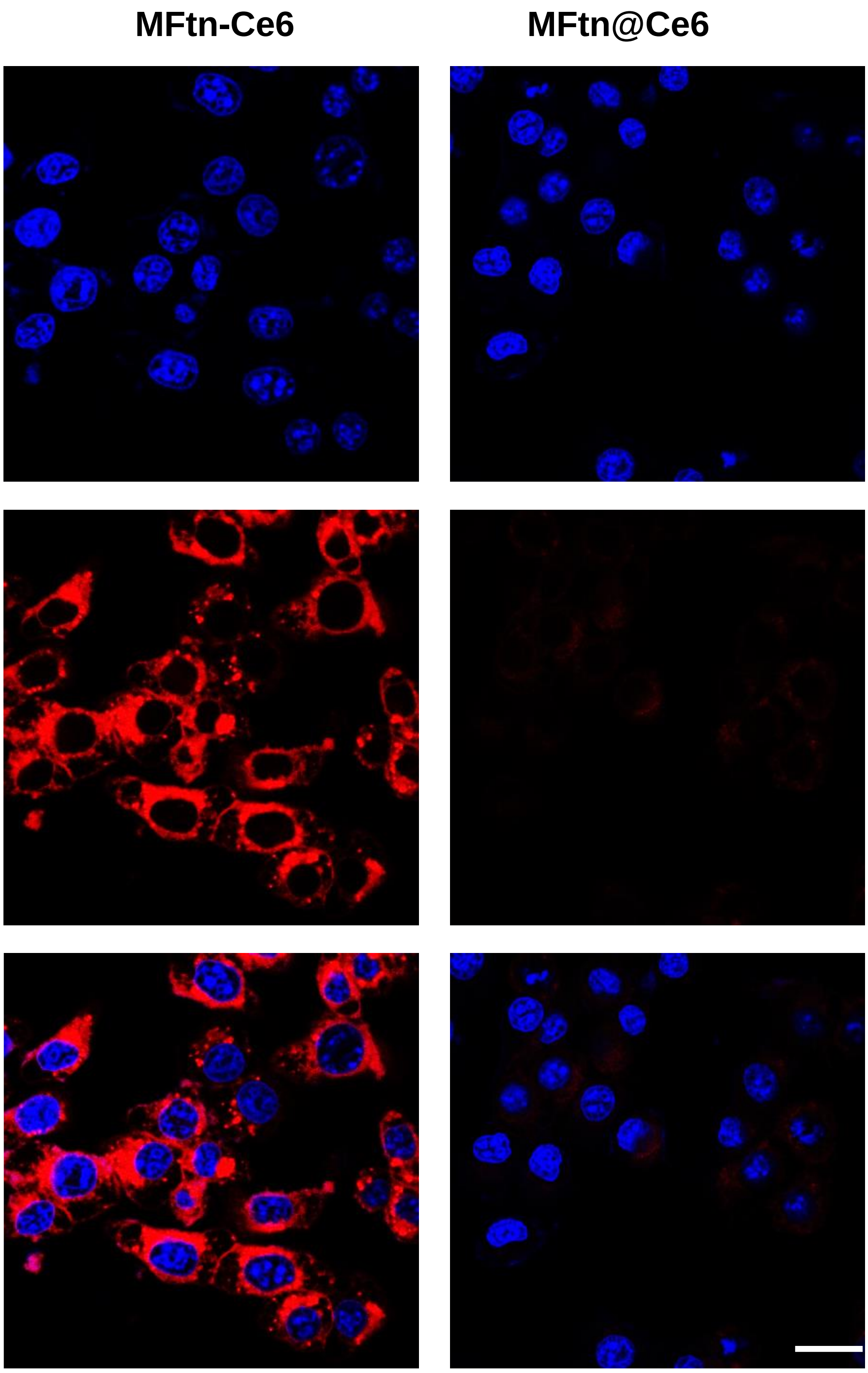

Figure S8. The cellular uptake of MFtn-Ce6 and MFtn@Ce6 at 12 hours. (The concentration of Ce6 was 20 μg mL^{-1}) Scale bar: 20 μm.

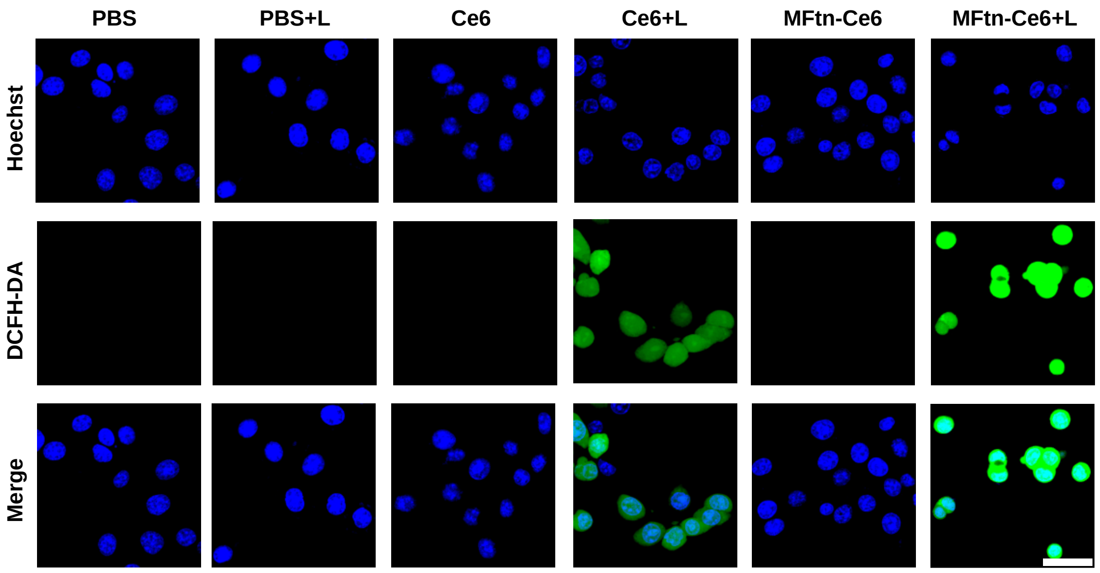

Figure S9. ROS generation in 4T1 cells incubated with PBS, free 2 μg/mL Ce6 and MFtn-Ce6. (660 nm laser, 25 mW/cm^2, 5 min). Scale bar: 20 μm.

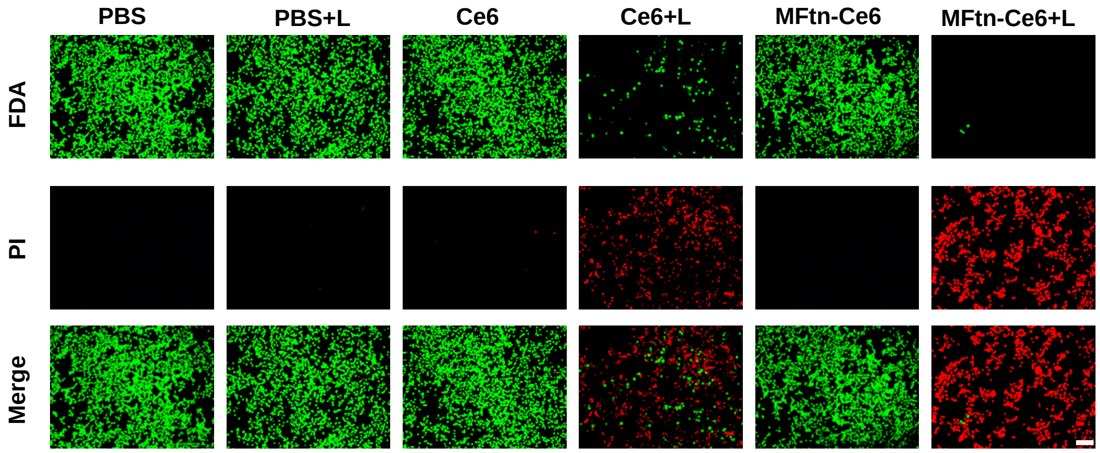

Figure S10. Live/dead cell assay for cells treated with PBS, free 2 μg/mL Ce6 and MFtn-Ce6. (FDA/green: live cells, PI/red: dead cells). Scale bar: 100 μm. Laser (660 nm laser, 25 mW/cm^2, 5 min)

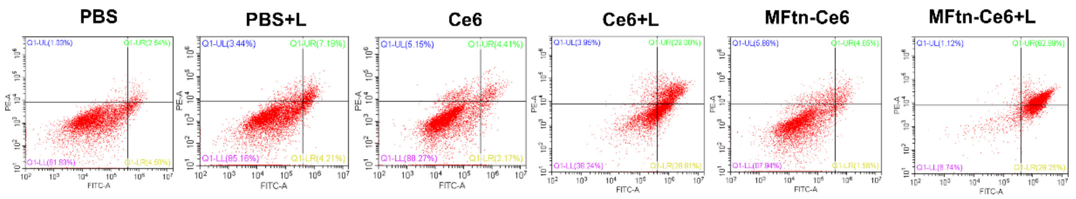

Figure S11. Flow cytometric analysis cell apoptosis. PBS, free 2 μg/mL Ce6 and MFtn-Ce6 with and without laser (660 nm laser, 25 mW/cm^2, 5 min)

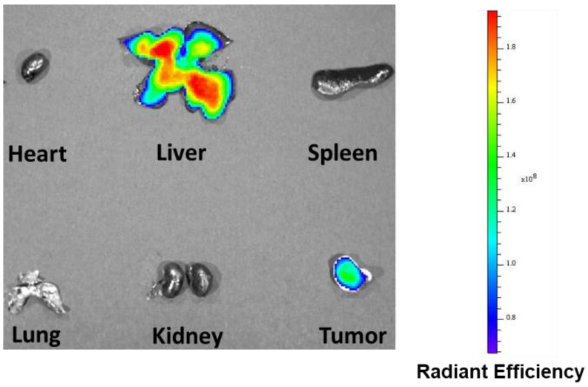

Figure S12. Ex vivo fluorescence images of the major organs and tumors at 48 h post injection of MFtn-Ce6.

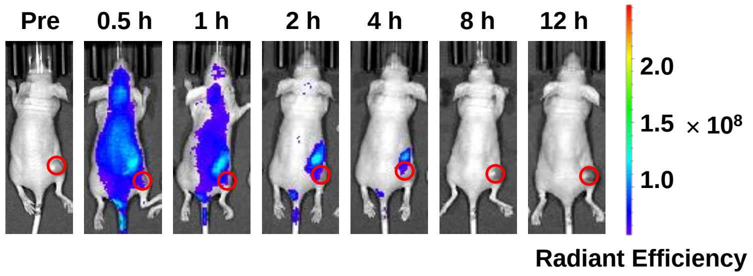

Figure S13. Real-time bio-distribution images of free Ce6 after injection. The tumor site is circled in red. (Total dose: 200 μL, 0.2 mg/mL Ce6).

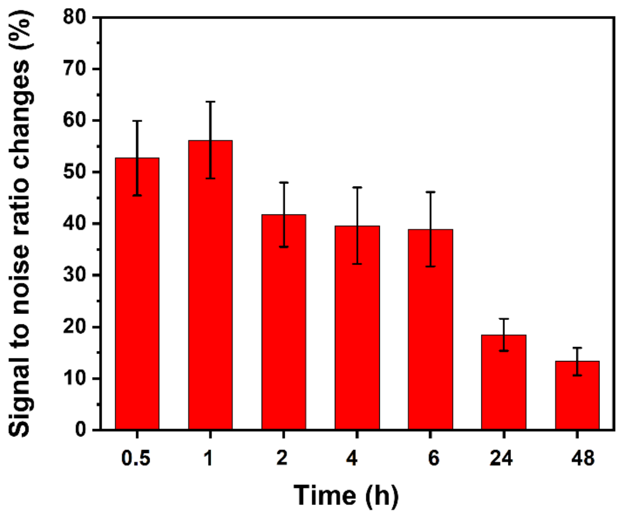

Figure S14. Time-dependent change of signal-to-noise ratio (ΔSNR) in kidney of *in vivo* T_2-weighted MR images after injection of MFtn-Ce6.

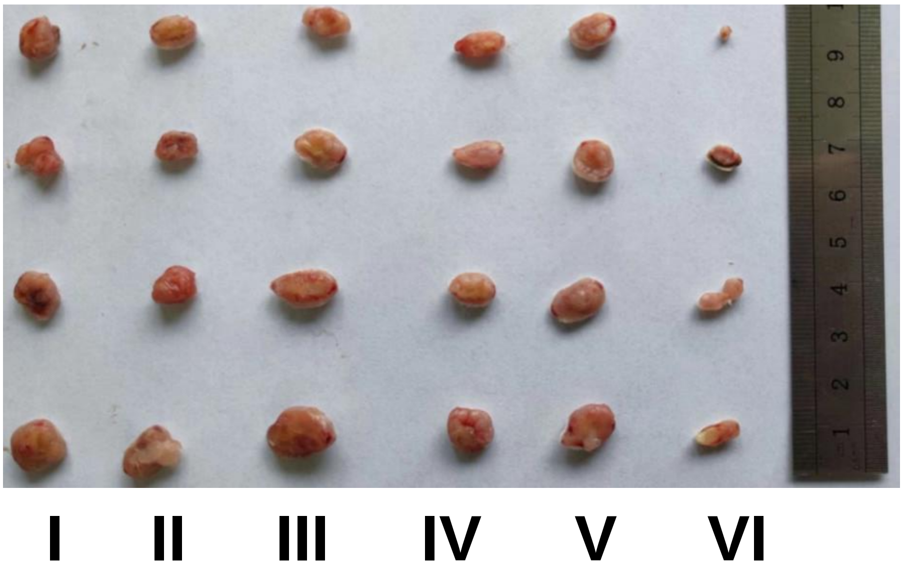

Figure S15. The xenograft tumors removed from mice after 14 days of treatment. I (PBS), II (PBS plus laser), III (Ce6), IV (Ce6 plus laser) V (MFtn-Ce6) VI (MFtn-Ce6 plus laser). n = 4

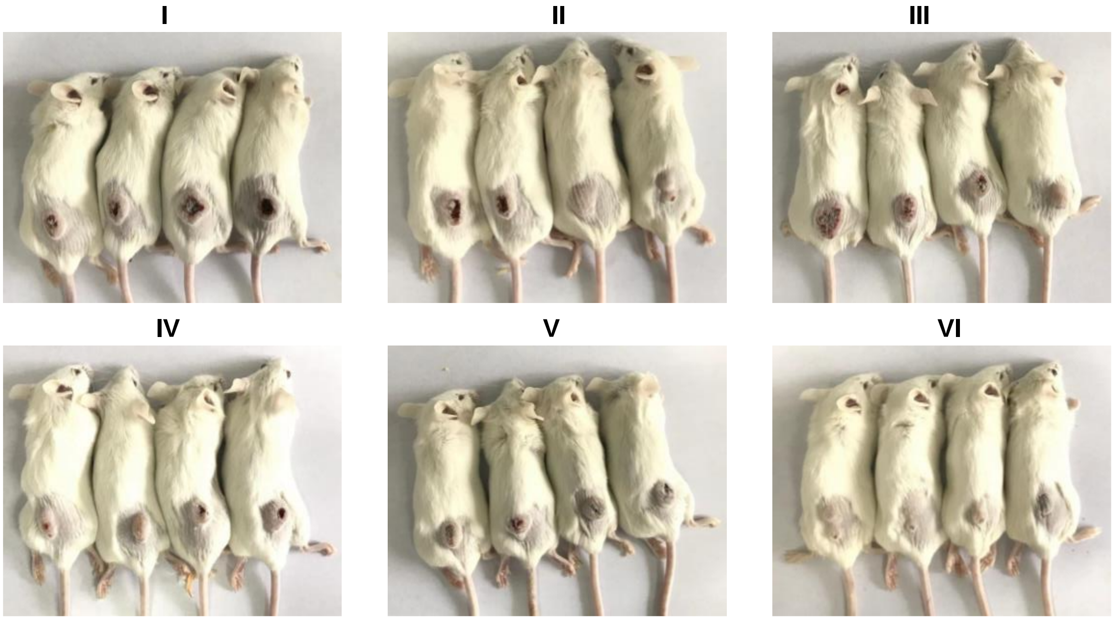

Figure S16. Photographs of these mice after 14 days of treatment. n = 4.

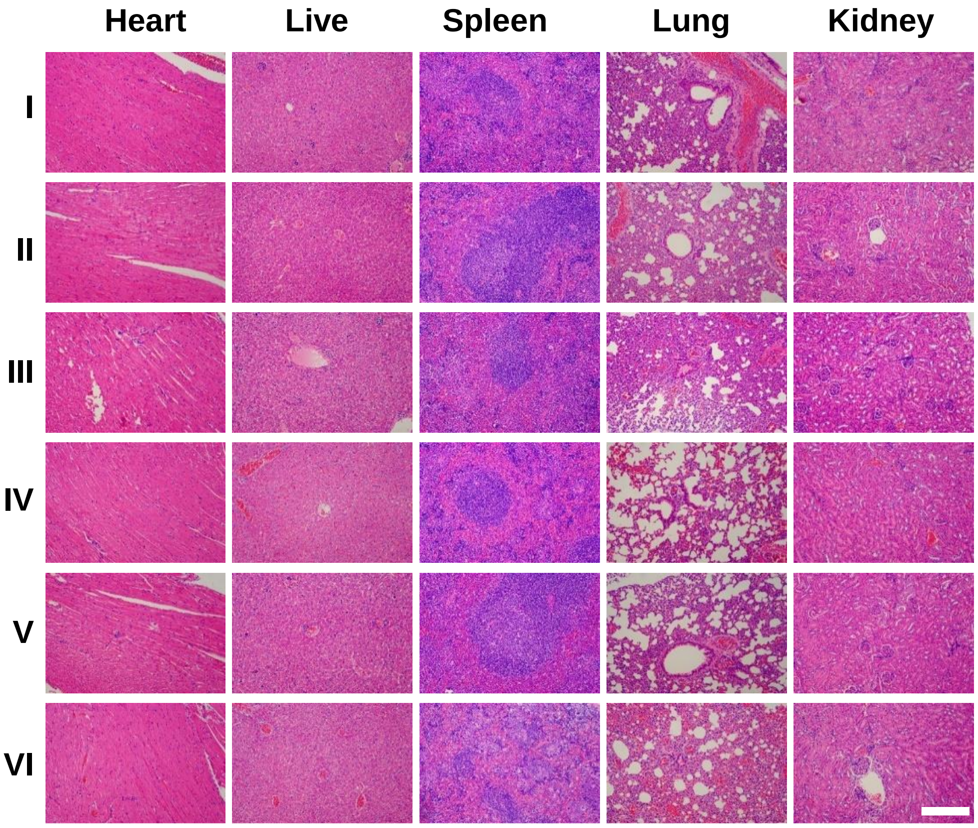

Figure S17. Pathological H&E-stained tissue sections of heart, liver, spleen, lung, and kidney, excised from mice at 14th day after various treatments. I (PBS), II (PBS plus laser), III (Ce6), IV (Ce6 plus laser) V (MFtn-Ce6) VI (MFtn-Ce6 plus laser). Scale bar: 100 μm.

Table S1. The summary of encapsulation efficiency, drug-loading rate of MFtn-Ce6 platform.

The concentration of Ce6	Encapsulation efficiency	The number of Ce6	Drug-loading rate of MFtn-Ce6
20 μg/mL	96.3 ± 4.5%	6.46 ± 0.30	0.76 ± 0.035%
40 μg/mL	92.1 ± 1.9%	12.35 ± 0.25	1.45 ± 0.029%
60 μg/mL	86.8 ± 2.7%	17.45 ± 0.55	2.04 ± 0.063%
80 μg/mL	73.7 ± 1.8%	19.76 ± 0.51	2.30 ± 0.058%
100 μg/mL	71.7 ± 2.9%	24.03 ± 0.96	2.79 ± 0.109%

(n = 3. Three parallel samples per group)

Reference

1. Liu, M.; Zhu, Y.; Wu, T.; Cheng, J.; Liu, Y., Nanobody-Ferritin Conjugate for Targeted Photodynamic Therapy. *Chemistry* **2020,** *26* (33), 7442-7450.
